# Finding my faith

**DOI:** 10.4103/0019-5545.44914

**Published:** 2009

**Authors:** Amandeep Sandhu

**Affiliations:** 159, 17th Cross, 30th Main, 6th Phase, J P Nagar, Bangalore – 560078, India

**Figure:**
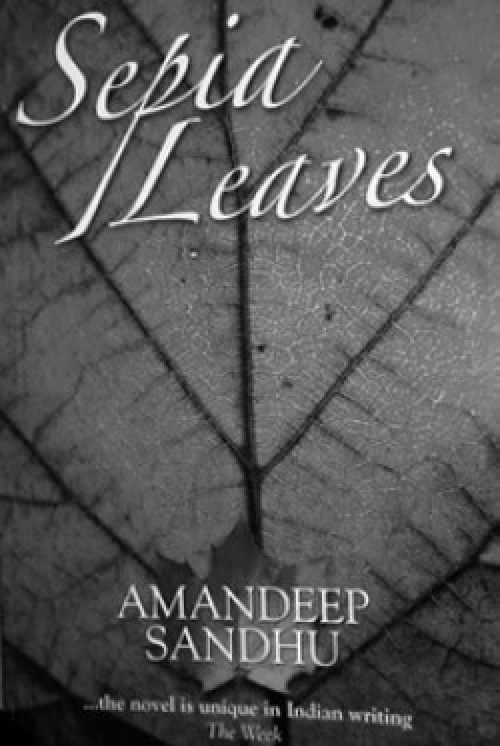


Readers tend to think that I had a difficult childhood and Sepia Leaves is a tale of survival. That is right, as a family we were huddled under a tree that was barren and which swayed in the other direction when the wind blew. My childhood was plagued with a mental illness where one person in our small family defied the normal rules of logic and behaviour. We put a name to the illness – Schizophrenia; and as I grew up I started recognizing the effect of that word in our lives.

After I finished school I sat for an all India medical entrance exam, but got up without completing it. The reason was all through my childhood and early teens my mother had been a full-blown case of schizophrenia: violent, moody, unstable, hallucinating, and uncommunicative. As per the wisdom of medical practices at the time and how much of it had percolated in a small town in East India all that the doctors had done was drug her, make her more a vegetable (Mineral and Eskazine and electric shocks from time to time). I was angry about losing my mother and was unhappy with a medical practice that had taken her further away from me. I had felt cheated by psychiatrists, it was as if I was drowning at high sea and the one plank that I could cling to had turned out hollow.

In the 1990s my father took my mother to her original psychiatrist who prescribed Trinicalm and Loxapac. They seemed to work but it was already too late, I was twenty years old and my thoughts had formed. I was anti-psychiatry and set loose in the world trying to find my faith. Then a series of events happened which made me realize that the problem was not with medication but with perception, with the way we had dealt with the problem: the society had stigmatized us, the doctors had provided myopic drug supply, and we ourselves wallowed in self-pity.

Through years of living alone with my mother and finding his own dreams coming to naught my father started getting depressed. Many years before he came to Bangalore he often cried alone in his room and once even fainted in the storeroom of his earlier house but my mother did not notice it. Over the last few years before his death my father became depressed but my mother's illness was so bright that my father's was overshadowed. You cannot have a small lamp next to a big sun.

When my parents came to Bangalore and my father showed signs of depression my mother was getting another problem but that got sidelined because all my focus was on my father. I do not know whether not going in for stent after he had an angina and we had discovered his blocked arteries, was a good decision (doctors had asked us to heal through change in diet and exercise), but only after he passed away and mother fainted that I realized that she was cardiomyopathic – an enlarged heart. The doctors gave her six months but she lived for four years. When she died it was out of breast cancer and her cardiologist, who was examining her heart over the years, could not notice a growth in her right breast, six inches away from his area of inspection.

During this period of giving care, for various reasons I myself went through depression but by now I had discovered that psychiatrists are not evil people. Thanks to a wonderful psychiatrist who counseled my father, my mother, and even me. Who took care to understand our whole family and our limitations. I took medication and recovered, my mother stabilized, my father had already passed away. I have come to realize that all doctors work under trying conditions and medical science has not found all the answers yet. But we all can find kindness in ourselves, like my psychiatrist did. What is needed is a world where people need not be roles but human beings, where we can have a little extra love to go around, a little care built into the system, a little assurance in our dealings, a little smile in the face of adversity.

Sepia Leaves makes a plea against apathy and in favour of compassion. Each illness is hard, each needs to be treated, but we are not all Arjuna looking at the eye of the bird that we want to surgically remove. As doctors we must realize that we are only one vertex of the pyramid, the patient being the other. The third vertex, the caregiver, is important, very important if the pyramid of health care has to stand tall.

A doctor wrote to me after she read the book. She said: I am a doctor who wants to pursue Psychiatry. I have worked in Psychiatry and have taken care of schizophrenics. Your book was an eye-opener. I always thought that I was a compassionate doctor who wanted to bring peace to the troubled minds. The very word Paagal infuriates me and I have always tried to convince people that every patient of Psychiatry can be made to have a normal social life with the help of medication, counseling, etc. But, I never saw beyond the patients. I never saw what it did to the families. All I was bothered was about making sure that the patient took the right prescription, came for regular follow-ups and went back to being normal till the relapse occurred.

At the peak of my mother's illness I went to all her doctors and my doctor friends with the list of medicines that my mother had to take. There were 28 items on the list. I asked each doctor if we could somehow reduce the tablets. No one was willing to reduce a single tablet. I recognize that each prescription is necessary but can we make drugs that combine treatment for multiple illnesses? A cousin of mine said, “28 pills! My, that is a full lunch.”

It is. Does a doctor have a slightly bigger point of view than his own super specialty? A holistic view? Does a doctor have more time to spend on a patient instead of treating us like irate customers at a city bus station?

When our psychiatrist read the draft of the book he said that this book had one of the most accurate depictions of the illness. In literature the mad becomes the other but my mother was not an other to me. What happened to her was not beyond the realm of my intuition. Of course, the understanding of her illness came much later and came through other experiences including trying to understand her life by writing about it and by living with her, taking care of her. A teacher of mine once said, “Poets are autistic people who have language.” Sepia Leaves is my attempt to find a holistic voice.

My father always referred to me as amanat, trust, that he had received from God. He saw it as his responsibility that the keepsake, me, is preserved. I, in turn, have thought of their lives with me as an amanat and have captured it in a book.

I have tried to find my faith in the struggle of an ordinary family, in the fact that as human we are all fallible but with some care we might be able to become whole. Sepia Leaves is a son's tribute to his mother who no one understood and a father who was too noble for his own good.

[*Editor's note: The review of the book by Dr. Alok Sarin appears on pg 71 of this issue*]

